# Supplementary material for: Development of a Recycling Process and Characterization of EVA, PVDF, and PET Polymers from End-of-Life PV Modules
Source: Materials (Basel). 2024 Feb 8;17(4):821. doi: 10.3390/ma17040821 (PMC10890113; doi:10.3390/ma17040821)
Supplement: Supplementary file 1 [file materials-17-00821-s001.zip › materials-2842574-supplementary.pdf]

materials

Supplementary Materials

Development of a Recycling Process and Characterization of EVA, PVDF and PET Polymers from End-of-Life PV Module

Marek Królikowski [1,*], Michał Fotek [2], Piotr Żach [2] and Marcin Michałowski [3]

[1] Faculty of Chemistry, Warsaw University of Technology, Noakowskiego 3, 00-664 Warsaw, Poland
[2] Faculty of Automotive and Construction Machinery, Warsaw University of Technology, Narbutta 84, 02-524 Warsaw, Poland; michal.fotek@pw.edu.pl (M.F.); piotr.zach@pw.edu.pl (P.Ż.)
[3] Faculty of Mechatronics, Warsaw University of Technology, św. Andrzeja Boboli 8, 02-525 Warsaw, Poland; marcin.michalowski@pw.edu.pl
* Correspondence: marek.krolikowski@pw.edu.pl; Tel.: +48-22-234-7475

Citation: Królikowski, M.; Fotek, M.; Żach, P.; Michałowski, M. Development of a Recycling Process and Characterization of EVA, PVDF and PET Polymers from End-of-Life PV Module. *Materials* **2024**, *17*, 821. https://doi.org/10.3390/ma17040821

Academic Editor: Xuming Xie

Received: 10 January 2024
Revised: 1 February 2024
Accepted: 5 February 2024
Published: 8 February 2024

Copyright: © 2024 by the authors. Submitted for possible open access publication under the terms and conditions of the Creative Commons Attribution (CC BY) license (https://creativecommons.org/licenses/by/4.0/).

Table S1. Density, *d* and dynamic viscosity, *η* of toluene solution during delamination PV module, measured at T = 298,15 K and p = 100 kPa.

Delamination/%	d/g·cm^{-3}	η/mPa·s
0 (pure solvent)	0.86231	0.553
20	0.86253	0.581
40	0.86265	0.610
50	0.86277	0.638
60	0.86283	0.668
80	0.86298	0.702
100	0.86315	0.768

Standard uncertainties are $u(T)$ = 0.05 K, $u(p)$ = 2 kPa, $u(d)$ = 5 × 10^{-4} g·cm^{-3}, $u_r(\eta)$ = 0.03 mPa·s.

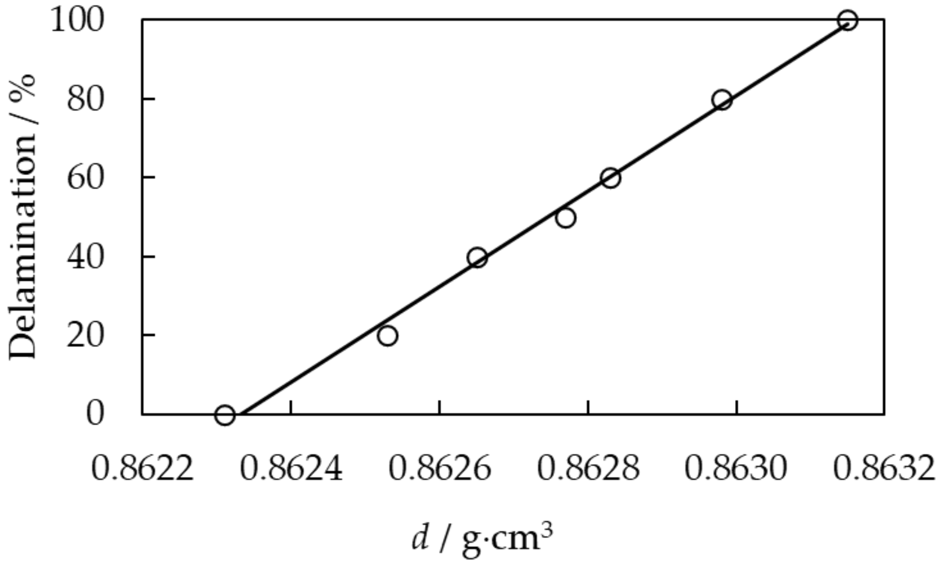

Figure S1. PV module delamination (%) as a function of the density toluene solution.

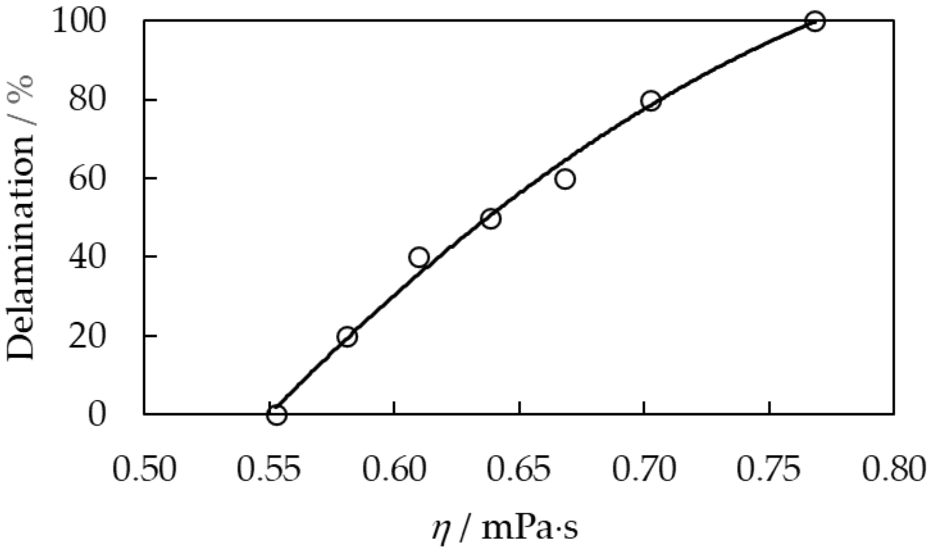

Figure S2. PV module delamination (%) as a function of the dynamic viscosity toluene solution.
